# Job requirements compared to medical school education: differences between graduates from problem-based learning and conventional curricula

**DOI:** 10.1186/1472-6920-10-1

**Published:** 2010-01-14

**Authors:** Christopher L Schlett, Hinnerk Doll, Janosch Dahmen, Ole Polacsek, Gero Federkeil, Martin R Fischer, Fabian Bamberg, Martin Butzlaff

**Affiliations:** 1 Institute for Teaching and Educational Research in Health Sciences, Private University Witten/Herdecke, Witten, Germany; 2 Cardiac MR PET CT Program, Massachusetts General Hospital and Harvard Medical School, Boston MA, USA; 3 Centre for Higher Education Development, Gütersloh, Germany; 4 Department of Clinical Radiology, University Hospitals Munich, Ludwig-Maximilians-University, Munich, Germany; 5 Scientific Director, Board of Directors, Private University Witten/Herdecke, Witten, Germany

## Abstract

**Background:**

Problem-based Learning (PBL) has been suggested as a key educational method of knowledge acquisition to improve medical education. We sought to evaluate the differences in medical school education between graduates from PBL-based and conventional curricula and to what extent these curricula fit job requirements.

**Methods:**

Graduates from all German medical schools who graduated between 1996 and 2002 were eligible for this study. Graduates self-assessed nine competencies as required at their day-to-day work and as taught in medical school on a 6-point Likert scale. Results were compared between graduates from a PBL-based curriculum (University Witten/Herdecke) and conventional curricula.

**Results:**

Three schools were excluded because of low response rates. Baseline demographics between graduates of the PBL-based curriculum (n = 101, 49% female) and the conventional curricula (n = 4720, 49% female) were similar. No major differences were observed regarding job requirements with priorities for "Independent learning/working" and "Practical medical skills". All competencies were rated to be better taught in PBL-based curriculum compared to the conventional curricula (all p < 0.001), except for "Medical knowledge" and "Research competence". Comparing competencies required at work and taught in medical school, PBL was associated with benefits in "Interdisciplinary thinking" (Δ + 0.88), "Independent learning/working" (Δ + 0.57), "Psycho-social competence" (Δ + 0.56), "Teamwork" (Δ + 0.39) and "Problem-solving skills" (Δ + 0.36), whereas "Research competence" (Δ - 1.23) and "Business competence" (Δ - 1.44) in the PBL-based curriculum needed improvement.

**Conclusion:**

Among medical graduates in Germany, PBL demonstrated benefits with regard to competencies which were highly required in the job of physicians. Research and business competence deserve closer attention in future curricular development.

## Background

The framework of medical education has always been a controversial subject. In 1899, Sir William Osler claimed that the complex and growing structure of medical knowledge was associated with difficulties in imparting the necessary skill set for a well-trained physician. Osler concluded that one way to overcome this issue may be to allow more time for independent study [1], today known as Problem-based Learning (PBL).

PBL is a method of knowledge acquisition whereby small groups of students, guided by tutors, develop their own learning objectives based on real-world case scenarios and then reconvene after a certain period of independent study to solve "the case" with their newly acquired medical knowledge [2,3]. There are several studies indicating that PBL is associated with a higher gain in medical competencies, in particular in interpersonal and cognitive domains [4-11]. The cognitive domain involves the development of intellectual skills to enable judgments about the value of ideas or materials; the interpersonal domain focuses on people interactions [12]. Prince et al. report in their study of 1,159 Dutch graduates from five medical schools with an average follow-up of 18 months after graduation that 83% of PBL-graduates are satisfied as regards their training in communication skills (such as interaction with patients or co-operation with other health professionals) compared to 41% of non-PBL graduates [8].

In contrast, other data shows that PBL may be associated with a deficiency in profession-relevant knowledge and writing skills regarding reports or articles [2]. Profession-relevant knowledge was defined in this context as knowledge and understanding of the scientific basis, etiology, pathogenesis and clinical manifestations of diseases as well as their current treatment options [13]. Similarly, Cohen-Schotanus et al. evaluate differences in clinical and general competencies and career development between PBL-based and conventional curricula in the Netherlands. In their sample of 294 subjects, they assess the appreciation of the curriculum, self-assessed medical competencies, time gap until residency placement and research output within three to six years after graduation. In a multivariate analysis, no differences for objective measures were found [14].

There are also a number of studies and long-term reports demonstrating a significant outcome benefit of PBL-based curricula. These findings are summarized in a systematic review by Koh et al. in 2008 [15]. Initially 102 articles were identified, but only 13 of these met the inclusion criteria of controlled trials, which evaluated the benefits of PBL as a teaching method in undergraduate medical education with additional assessment of graduate physicians. The authors conclude that PBL has a positive effect on most physicians' competencies after graduation, mainly with respect to social (including emotional and moral aspects) and cognitive dimensions [15].

However, no study has yet described the self-perceived relevance of key competencies for fulfillment of day-to-day work, how well theses competencies are taught and how well medical school education prepared for job requirements between graduates from PBL-based and conventional curricula in Germany. We hypothesized that PBL-curricula actually stimulate independent learning and demonstrate equivalent or superior professional competencies compared with graduates of more traditional curricula [2,8,16].

The primary research question of our study was to assess and compare graduates from PBL-based and from conventional curricula as regards to nine key competencies required at their day-to-day work. Secondly, we wanted to assess and compare how well those competencies were taught in medical school between those two groups of graduates. And finally we evaluated whether these competencies as taught in medical school sufficiently cover the day-to-day work requirements of medical graduates.

## Methods

### Study population

We studied graduates of German medical schools who graduated between 1996 and 2002 using a standardized survey carried out by the independent Centre for Higher Education Development (CHE, Germany). All 37 German medical schools were contacted and questionnaires were sent out to all graduates. Importantly, there was only one medical school between 1996 and 2002 offering a complete PBL-based curriculum in Germany [17,18]. This medical school at the private University Witten/Herdecke was founded in 1983 with the major goal to provide an alternative in education with self-guided, patient-oriented medical training throughout medical school [18].

The survey was conducted between 2004 and 2005; details of the study procedure are provided elsewhere [19]. In brief, the CHE contacted medical graduates by mail. A link to the online questionnaire was given in individually addressed covering letters by the CHE. Graduates were asked to carry out their self-assessment online. An anonymized ID-code for each graduate prevented multiple survey responses. Contact addresses were obtained from the database of the State Medical Chambers in Germany and from alumni associations wherever applicable. According to an official request to the institutional ethics committee of the University Witten/Herdecke, approval by an ethics commission was not necessary for conducting the study, as there was no intervention. All participants in the study participated voluntarily and provided informed consent.

### Outcome measures

As an independent study core, the CHE developed the standardized questionnaire which consisted of 35 items [19]. The questionnaire contained baseline demographic data and professional status. Furthermore, the questionnaire assessed nine competencies as required by current employment and whether these were adequately represented in the respective curriculum (as translated from the German original: "Please evaluate to which extent the following competencies are (A) required in your day-to-day job and (B) were taught in medical school"). The design of the text questions was based on reports describing the general skills necessary for professional practice in medicine [13,20,21]. The following nine competencies were included to be evaluated by the graduates: "Independent learning/working", "Practical medical skills", "Psycho-social competence", "Teamwork", "Problem-solving skills", "Medical knowledge", "Interdisciplinary thinking", "Business competence", and "Research competence". These outcome variables were collected using a 6-point Likert scale on which a score of six (6) reflects the positive end ["very intensively required/taught"], and a score of one (1) the negative end ["not at all required/taught"] of the scale. A comparison of the final national board examination grades was performed based on the publications of the German Institute for Medical and Pharmaceutical Exam Questions [22].

### Statistical analysis

Baseline demographics and clinical characteristics are presented as mean ± standard deviation (SD) for continuous variables and percentage [frequency] for categorical variables. Outcomes of interest were the self-assessed competencies as reported by graduates of German medical schools. Graduates were stratified by whether their medical curriculum was PBL-based (University Witten/Herdecke) or conventional (all other German medical schools). To ensure generalizabilty, universities were excluded if less than 50 of their graduates responded.

Differences in baseline demographics between the two stratified cohorts were determined using chi-square and two-tailed t-tests for categorical and continuous variables, respectively. Fisher's exact test was used if subgroups included less than ten subjects.

**Figure 1:**
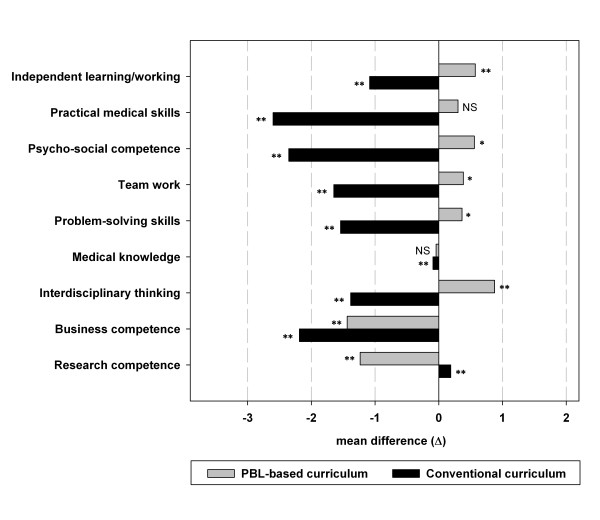
**Differences between required competencies at the day-to-day work and competencies taught in medical school**. Figure 1 illustrates a subtraction for of the both ratings, how far those competencies were required at the day-to-day work and how well those were taught in medical school. Negative mean differences indicate a deficit in the teaching of competencies as compared to job requirements; positive mean differences indicate a surplus. The competencies were sort from top to the bottom by their relevance at the day-to-day work of physicians. NS denotes no significant difference, * differences with a p-values < 0.05 and ** differences with a p-values < 0.001.

To determine whether medical school education sufficiently covers the job requirements of physicians, we calculated the mean difference (Δ) between the ratings of competencies as required at the day-to-day work and as taught in medical school by subtracting those from each other. This comparison demonstrates whether an education is perceived to cover job requirements (Figure 1). It also permitted detection of deficits (mean difference [Δ] was negative) and surplus (mean difference [Δ] was positive) of teaching in the different curricula for each competence. To evaluate whether those so calculated deficit or surplus for a competence was significant, we applied a paired two-tailed t-test (Figure 1).

To express effect size, Hedges' g was computed for all significant differences between graduates from the PBL-based curriculum and from the conventional curricula regarding the research questions "Required competencies at the day-to-day work", and "Competencies as taught in medical school" [23]. A Hedges' g < 0.5 was defined as a "small", 0.5 to 0.8 as a "moderate" and >0.8 as a "large" effect size [24].

All analyses were performed using SPSS (Version 13, SPSS Inc., Chicago, IL, USA) and SAS (Version 9.2, SAS Institute Inc., Cary, NC, USA). A p-value < 0.05 was considered to indicate statistical significance.

## Results

Overall, more than 35,000 graduates of all 37 German medical schools were contacted. Among these, three universities were excluded from the analysis due to low response rates (less than 50 responses of graduates per medical school for the University of Rostock, the University of Greifswald, and the University of Regensburg). Thus, completed questionnaires of 4,821 graduates of 34 universities formed the study sample (14%). Of these, 101 were graduates of the PBL-based medical school curriculum and 4,720 were graduates of conventional medical curricula. The response rate was significantly higher in the group of PBL-graduates (53% vs. 14% of the conventional curricula respectively; p < 0.001).

Baseline demographics of the two groups are detailed in Table 1. There was no difference with respect to gender (p = 0.89), total study time (p = 0.80), proportion of graduates with medical degree thesis (p = 0.08), proportion of graduates with other degrees (p = 0.91), choice of first job location (categorized in "University Hospital", "General Hospital", "Private Practice", "other") and current job condition (categorized in "Freelance", "Open-ended contract", "Limited contract" and "Temporary work") between the two groups. Further, both groups were similar with respect to work experience (>2 years, 79% vs. 73%, p = 0.16, PBL-based vs. conventional curricula, respectively). In contrast, graduates of the PBL-based curriculum were in average older, had spent significantly more time abroad, and had undergone more frequent clinical training subsequent to medical school graduation (all p < 0.001; Table 1). Also, graduates of the PBL-based curriculum had significantly better average grades in the final written examination (2.35 ± 0.2 vs. 2.46 ± 0.1, p = 0.02, PBL-based vs. conventional curricula, respectively).

**Table 1:** Baseline demographics and study characteristics

	*PBL-based Curriculum*	*Conventional Curriculum*	*p-value*
	N = 101	N = 4720	
Gender (female)	49% (49)	49% (2272)	0.89
Age at matriculation (mean)	22.2 years	21.4 years	<0.001
Duration of study (mean)	13.2 semester	13.3 semester	0.80
Studied abroad	91% (92)	60% (2801)	<0.001
Medical research thesis	54% (55)	63% (2974)	0.08
Another university degree	4% (4)	4% (189)	0.91
Started residency	89% (90)	71% (3351)	<0.001
First job at			0.52
University Hospital	29% (29)	27% (1178)	
General Hospital	60% (59)	65% (2888)	
Private Practice	5% (5)	4% (198)	
other	6% (6)	4% (172)	
Job experiences, >2 years	79% (80)	73% (3446)	0.16
Current labour condition			0.17
Freelance	2% (2)	<1% (24)	
Open-ended contract	12% (12)	10% (463)	
Limited contract	86% (83)	89% (4206)	
Temp work	0% (0)	<1% (28)	

Baseline demographics and study characteristics between graduates of the PBL-based and conventional medical curriculum among German medical schools.

### Required competencies at the day-to-day work of physicians

Overall, graduates of both curricula rated the job requirements similarly. The most required competencies at their day-to-day work were "Independent learning/working", "Practical medical skills", and "Psycho-social competence" (5.19 ± 0.9, 5.17 ± 1.2, and 4.89 ± 1.3, respectively; Table 2). In contrast, differences between the two groups were found with respect to "Research competence" and "Medical knowledge". Graduates of the PBL-based curriculum rated the "Research competence" as a significantly higher job requirement as compared to graduates of conventional curricula (3.78 ± 1.6 vs. 2.92 ± 1.5, p < 0.001; respectively). Conversely, graduates of the PBL-based curriculum rated "Medical knowledge" as less required compared to graduates of conventional curricula (4.43 ± 1.2 vs. 4.78 ± 1.0, p = 0.02; respectively). However, both differences demonstrated only a small to moderate effect size, while Hedges' g for differences in "research competence", and "medical knowledge" were 0.6 and 0.4, respectively (Table 2).

**Table 2:** Evaluation of required competencies at the day-to-day work of physicians

	*PBL-based Curriculum*	*Conventional Curriculum*	*p-value*	*Effect size*
Independent learning/working	5.01 ± 1.6	5.19 ± 0.9	0.37	---
Practical medical skills	5.08 ± 1.6	5.17 ± 1.2	0.68	---
Psycho-social competence	4.83 ± 1.7	4.89 ± 1.3	0.90	---
Team work	4.91 ± 1.6	4.84 ± 1.1	0.43	---
Problem-solving skills	4.86 ± 1.5	4.81 ± 1.0	0.59	---
Medical knowledge	4.43 ± 1.2	4.78 ± 1.0	0.02	small
Interdisciplinary thinking	4.56 ± 1.5	4.17 ± 1.1	0.39	---
Business competence	3.55 ± 1.4	3.53 ± 1.6	0.92	---
Research competence	3.78 ± 1.6	2.92 ± 1.5	<0.001	moderate

Results for each group were expressed as mean ± standard deviation on this 6-point Likert scale, where "6" indicated "very intensively", and "1" indicated "not at all" required at the day-to-day work of physicians. For significant differences, a small, moderate, and large effect size was defined as Hedges' g <0.5, 0.5-0.8, and >0.8 respectively.

### Competencies as taught in medical school

Assessment of the competencies as taught in medical school differed significantly between the two groups (Table 3). As such, graduates of the PBL-based curriculum deemed that their curriculum facilitated preparation in "Independent learning", "Practical medical skills", "Psycho-social competence", "Teamwork", "Problem-solving skills", and "Interdisciplinary thinking", whereas graduates of the conventional curriculum rated their teaching as consistently inferior (Table 3, all p < 0.001). The greatest effect size for the differences in ratings between the two groups were observed for "Psycho social competence" (Hedges' g: 2.4) and "Practical medical skills" (Hedges' g: 2.4), followed by "Interdisciplinary thinking" (Hedges' g: 1.9). In contrast, PBL graduates rated "Medical knowledge" and "Research competence" inferior compared to graduates from conventional curricula (4.38 ± 1.0 vs. 4.69 ± 0.9, p = 0.002, and 2.57 ± 1.3 vs. 3.10 ± 1.3, p < 0.001 respectively). However, the difference for these two competencies demonstrated only a small effect size (Hedges' g: 0.3 and 0.4 for "Medical knowledge" and "Research competence", respectively).

**Table 3:** Evaluation of competencies as taught in medical school

	*PBL-based Curriculum*	*Conventional Curriculum*	*p-value*	*Effect size*
Independent learning/working	5.61 ± 0.7	4.11 ± 1.3	< 0.001	large
Practical medical skills	4.38 ± 0.7	2.59 ± 1.2	< 0.001	large
Psycho-social competence	5.45 ± 0.7	2.54 ± 1.3	< 0.001	large
Team work	5.33 ± 0.9	3.19 ± 1.3	< 0.001	large
Problem-solving skills	5.26 ± 0.8	3.28 ± 1.2	< 0.001	large
Medical knowledge	4.38 ± 1.0	4.69 ± 0.9	0.002	small
Interdisciplinary thinking	5.44 ± 0.7	3.33 ± 1.1	< 0.001	large
Business competence	2.14 ± 1.2	1.35 ± 0.7	< 0.001	large
Research competence	2.57 ± 1.3	3.10 ± 1.3	< 0.001	small

Ratings are provided as mean ± standard deviation on a 6-point Likert scale, where "6" indicated "very intensively", and "1" indicated "not at all" taught in medical school. For significant differences, a small, moderate, and large effect size was defined as Hedges' g <0.5, 0.5-0.8, and >0.8, respectively.

"Business competence" was rated to be taught worse than any other competence, with similar results in both groups (4.86 ± 1 vs. 5.65 ± 1; Table 3); however, ratings of graduates of the PBL-based curriculum were significantly superior as compared to graduates of the conventional curriculum (p < 0.001).

### Differences between required at day-to-day work and taught in medical school

The differences between competencies required at the day-to-day work and competencies taught in medical school were illustrated for both groups in figure 1. The ratings given by graduates of the PBL-based curriculum indicated a significant deficit (negative values) in the teaching of "Business competence" (Δ - 1.44, p < 0.001) and "Research competence" (Δ -1.23, p < 0.001). In comparison, the ratings by graduates of the conventional curriculum indicated deficits in all competencies except for "Research competence" (Δ +0.19, p < 0.001). In contrast, significant surplus of medical school education (positive values) as compared to requirements at the day-to-day work were identified for the PBL-based curriculum in "Interdisciplinary thinking" (Δ +0.88, p < 0.001), "Independent learning/working" (Δ +0.57, p < 0.001), "Psycho social competence" (Δ +0.56, p = 0.003), "Teamwork" (Δ +0.39, p = 0.03), and "Problem-solving skills" (Δ +0.36, p = 0.02).

## Discussion

### Required competencies at the day-to-day work of physicians

Overall, both groups of graduates assess the value of core competencies as demanded by their day-to-day work similarly. Competencies such as "Practical medical skills" and "Psycho social competence" are related to high clinical value and mirror classic PBL strengths [2]. Interestingly, medical doctors overall rate "Independent learning/working" as a non-medical competence to be more required than other medical competencies. One could argue that these findings are in line with the theory introduced by Sir Osler in 1899 emphasizing the need to prepare for lifelong learning [1,25]. Although job requirements are similar for both groups, graduates from the PBL-based curriculum evaluate "Medical knowledge" as less required and "Research competence" as more highly required. While it is possible that PBL graduates are more used to compensating medical knowledge gaps by independently acquiring new information, this ability may be less developed/trained in graduates from a conventional curriculum. This hypothesis is in line with a recent study by Prince et al., which indicates that a lower rating of "possession of profession-relevant knowledge" is associated with PBL-based curricula [8]. However, those evaluated differences showed only a small to moderate effect size.

### Competencies as taught in medical school

The rating "How well were these competencies taught in medical school?" differs significantly between the two groups of graduates. The PBL-curriculum was rated superior in seven out of nine competencies, all with a large effect size. "Independent learning/working", "Psycho-social competence" and "Practical medical skills" are rated as the top three competencies that were acquired during PBL-based medical school education. These findings are similar to the current study of Koh et al., where the author demonstrates strong evidence that graduates from a PBL-based curriculum benefit especially in social and cognitive competencies [15]. Our results confirm the strength of PBL-based curriculum in teaching social and cognitive competencies. Furthermore, our results show that the reformed curriculum based on PBL not only affects the typical PBL-related competencies, but also more general work-related skills which have been shown to be important for success in the medical professions [2]. PBL-based graduates in particular rated "Practical medical skills" as being much better taught at medical school compared to their colleagues from conventional curricula. These findings may support the hypothesis that PBL methods directly and indirectly improve medical skills by improving independent learning and communication skills [2,8,26].

In contrast to the described self-perceived benefits of the PBL-based curriculum, the conventional curricula were rated better for teaching of "Medical knowledge" and "Research competence". Case-studies, as used in PBL-based curricula, are time consuming [4]. Further, systematic lectures are (partly) replaced by individual seminars where students can raise and work on their own questions. Both effects may lead to impartment less medical knowledge in a PBL-based curriculum as compared to a conventional curriculum in medical school [2]. However, no disadvantages were observed regarding performance in the state administered final national board medical examination, in fact graduates from the PBL-based curriculum graduated with better average results in our study cohort as compared to graduates from the conventional curriculum. Other studies comparing USMLE (United States Medical Licensing Examination) Step 1 and 2 mean scores and pass rates showed similar performance of graduates from PBL-based und conventional curricula in the United States [5]. Research competence may be another weakness of PBL-based curriculum. This finding was previously shown in a review by Colliver et al. [27]. This review in conjunction with our data leads us to the explanation that PBL-based curricula have a distinct disadvantage in losing the ties between educational theory and research [27].

### Differences between required competencies and competencies taught in medical school

By directly comparing "How much a competence is required at work?" and "How well this competence was taught in medical school?", the curricular performance can be estimated in relation to job requirements. Our data suggests that the PBL-based curriculum fulfilled all job requirements except for two competencies (see below) and moreover, the PBL-based provided a significant educational surplus in five competencies, especially in "Interdisciplinary thinking" and "Independent learning/working" (both p < 0.001, Figure 1).

Our results further demonstrate that "Business competence" is insufficiently taught in both curricula. Thus, it may be of great interest to incorporate aspects of business administration and management which apparently are demanded by the clinical job to enhance both curricula. In fact, in a study by Larson et al., the investigators demonstrate that implementation of joint MD/MBA programs at US universities has considerably increased from six programs in 1993 to 33 programs in 2002 [28]. In addition, "Research competence" is not sufficiently taught by the PBL-curriculum and needs further improvement to meet job requirements.

### Limitations

Our results need to be evaluated in the context of our study limitations. Most importantly, our findings may be limited by selection bias as our overall response rate was low, although we included nation-wide over 4,800 graduates. Furthermore, the response rate differed between both groups. While only one medical school in Germany (University Witten/Herdecke) offered a PBL-based curriculum during 1996 and 2002, there may be differences with respect to realization of the PBL framework which may limit external validity of the observed findings. Even so our data was collected 2004/05, it has still relevant for the development of medical education systems, especially while the major part of medical schools is still based on a conventional curriculum. In addition, PBL-based curriculum should be developed further under the consideration of the identified weaknesses. Finally, our assessment of competencies required at day-to-day work as well the competencies as taught in medical school was based on self-perception. Some studies suggest that self-perception can differ from objective measures [14,29]. In this context, an interesting aspect may be that the evaluation of job requirements is very similar, even so great differences with a large effect size are observed between the evaluation of PBL-based and conventional curricula.

Given the limitations of the present analysis, further research to confirm and extend our findings is warranted. It may be of additional value to approach and survey employers or professional colleagues about their perceived skills of PBL and conventional medical graduates. Moreover, to address the limitation of low response rates, it may be more feasible to use personal interviews rather than postal or online questionnaires. Finally, more medical schools should be included which offer PBL-based curricula today. Thus longitudinal follow-up studies and objective assessment of competencies are necessary to fully evaluate the impact of PBL on medical education.

## Conclusion

In summary, this large survey among medical graduates demonstrates similar self-perceived job requirements between graduates from PBL-based and conventional curricula. Nevertheless, the PBL curriculum is associated with a strong positive effect on all key competencies, which are highly required in the job of physicians. However, research and business competencies deserve closer attention in future curricular development.

## Abbreviations

CHE: Centre for Higher Education Development; PBL: problem-based learning; SD: standard deviation.

## Competing interests

The authors CLS, HD, JD and OP are medical students at the University Witten/Herdecke. The authors MB and MRF are employees of the University Witten/Herdecke.

## Authors' contributions

Authors CLS, HD, JD, OP, GF, and MB all contributed to the design of the study, data analysis, and drafting of the manuscript. Authors FB and MRF contributed to data analysis and interpretation, and critical revision of the manuscript. All authors have read and provided approval to the manuscript as submitted.

## Pre-publication history

The pre-publication history for this paper can be accessed here:

http://www.biomedcentral.com/1472-6920/10/1/prepub
